# Alcohol Consumption in Concomitant Liver Disease: How Much is Too Much?

**DOI:** 10.1007/s11901-017-0343-0

**Published:** 2017-04-22

**Authors:** Hannes Hagström

**Affiliations:** 1 Centre for Digestive Diseases, Division of Hepatology, Karolinska University Hospital, SE-141 86 Stockholm, Sweden; 2 Clinical Epidemiology Unit, Department of Medicine, Solna, Karolinska Institute, Stockholm, Sweden

**Keywords:** Alcohol, Cirrhosis, Fibrosis, NAFLD, Liver disease

## Abstract

**Purpose of Review:**

High consumption of alcohol can lead to cirrhosis. The risk of a low to moderate consumption of alcohol in the setting of a concurrent liver disease is less clear. The aim of this review is to sum the evidence on the risk of adverse outcomes in patients with liver diseases other than alcoholic liver disease who consume alcohol.

**Recent Findings:**

High alcohol consumption is strongly associated with adverse outcomes in most liver diseases. For hepatitis C, some evidence points to an increased risk for fibrosis progression also with low amounts. For non-alcoholic fatty liver disease, most studies indicate an inverse association between fibrosis and alcohol consumption, but methodological limitations reduce inference.

**Summary:**

High alcohol consumption is associated with an increased risk of fibrosis progression and other adverse outcomes, while less is clear regarding low to moderate consumption. Obtaining high-level evidence on this topic ought to be the objective of future studies. Currently, an individual risk profile should be obtained in patients with liver disease who consume alcohol.

## Introduction

Alcohol consumption is deeply embedded in most human societies, with some evidence suggesting that production of alcohol started as early as 10,000 BC [1, 2]. As an extremely available stimulant, the consumption of alcohol is vast, with 86% of US citizens reporting any lifetime alcohol consumption [3], and a mean consumption of 8.6 l of pure alcohol per year in the USA, compared to 6.2 l per year globally [4]. Alcohol has been reported to account for 85,000 deaths per year in the USA [5], and as much as 5.9% of all global deaths can be attributed to alcohol [4].

It has long been known that alcohol has detrimental effects on the liver by inducing alcoholic liver disease (ALD) [6], and alcohol accounts for up to 50% of all deaths in liver cirrhosis on a global scale [4]. However, although 90% of persons who consume more than 60 g of alcohol per day, roughly equivalent to six drinks, develop steatosis [7], significant fibrosis or cirrhosis only develops in up to 30% of this population [8–10]. In contrast, a low to moderate consumption of alcohol, below two drinks per day in women and three drinks per day in men, has not been associated with an increased risk for liver disease and is currently considered safe in most countries [11, 12], although a more restrictive approach is currently advocated in some countries, including the UK where the recommended maximum intake for men is now two drinks per day [12].

The role of low to moderate consumption in the setting of a concurrent liver disease is controversial, as an interactive effect could be present. This review briefly sums the epidemiological evidence for the risk of alcohol consumption in patients with liver diseases other than alcoholic liver disease.

## NAFLD

Non-alcoholic fatty liver disease (NAFLD) is strongly associated with obesity, insulin resistance, and the metabolic syndrome [13–15]. Tracing the global obesity pandemic, NAFLD is now the most common liver disease, affecting up to 25% of the global population [16•] and is expected to become the leading indication for need of liver transplantation in the USA in the near future [17, 18]. Epidemiological studies have suggested an interactive effect between at least high-grade consumption of alcohol and obesity. In a study of almost 10,000 British men followed for 29 years, an increased risk for death in liver disease was found for consumption of more than 15 drinks of alcohol per week across all BMI categories. However, this risk was accentuated in obese men (RR 18.9, 95% CI 6.84–52.4) compared to normal weight men (RR 3.16, 95%CI 1.28–7.80) [19]. Similar findings have been found for women in the Million Women Study [20], where women who consumed more than 150 g of alcohol per week had a higher risk for development of liver cirrhosis if they also were obese than if they had a normal BMI.

In contrast, in patients with known NAFLD and compared to abstainers, a low to moderate consumption of alcohol has been associated with lower values of ALT [21], lower prevalence of hepatic steatosis [22, 23•, 24, 25], non-alcoholic steatohepatitis (NASH) [26], carotid plaques [27], and lower stages of fibrosis [26, 28•]. This is of importance since the most significant predicting factor for mortality in NAFLD is the stage of fibrosis [29, 30]. Contrasting this, a recent Mendelian randomization study found that NAFLD patients with a mutation in the aldehyde dehydrogenase gene, which leads to a slower metabolism of ethanol and therefore making persons with the mutation less likely to consume alcohol due to more side effects, did not have higher stages of fibrosis or prevalence of NASH [31••].

Heavy episodic drinking has been shown to increase the risk of fibrosis progression in biopsy-proven NAFLD [32], although this study did not evaluate lifetime consumption. There have been no randomized controlled trials of alcohol consumption in patients with NAFLD. However, a RCT of moderate red wine consumption in healthy students found that consumption of 33 g per day in men and 16 g per day in women for 3 months did not induce MRI-measured steatosis in single subject, indicating that to develop steatosis, either a higher dose or a longer duration of alcohol consumption is needed to induce steatosis [33••].

Notably, the main cause of death in NAFLD is cardiovascular disease [29]. Epidemiological studies indicate a J-shaped association between the amount of alcohol consumed and the risk of cardiovascular disease [34, 35], indicating that a low to moderate consumption of alcohol might actually be beneficial in NAFLD. A more extensive review on the effect of low to moderate alcohol consumption on NAFLD was recently published for the interested reader [36•].

## Hepatitis C

Chronic viral hepatitis C (HCV) is highly prevalent, with data indicating that between 2.7 and 5.2 million persons are infected in the USA [37–39] and approximately 130 million cases globally [40]. HCV-related disease, including cirrhosis and hepatocellular carcinoma, accounts for around 700,000 deaths each year [41]. Alcohol consumption is high in many patients with HCV, especially those infected through intravenous drug use [42, 43]. A large sum of evidence points toward an interactive effect on the risk for fibrosis progression and development of cirrhosis in patients with HCV and a high consumption of alcohol of at least 30 g per day [44–47]. The role of low to moderate alcohol consumption in HCV is less clear. A French cross-sectional study of 260 HCV patients found no increased risk of fibrosis progression in subjects consuming below 30 g per day [48]. By contrast, in a group of 78 untreated patients with HCV that underwent paired liver biopsies with a median time of 6.3 years between biopsies, higher alcohol consumption and higher drinking frequency was independently associated with fibrosis progression. All these patients drank below 40 g of alcohol per day (median 4.8 g per day), suggesting that even a low consumption of alcohol is harmful in HCV [49].

## Hepatitis B

As for HCV, a high consumption of alcohol corresponding to more than 30 g/day is associated with adverse outcomes, including an increased risk for mortality [50] and development of hepatocellular carcinoma [51, 52]. Regarding low to moderate alcohol consumption, less is clear. In a study of 1045 Chinese patients with hepatitis B investigated with transient elastography, patients who reported consumption of 1–20 g of alcohol per day did not have advanced fibrosis to a higher extent than patients who reported being abstainers [53]. As in many other studies, these are cross-sectional data which is prone to potential misclassification bias due to under-reporting of alcohol consumption in heavy drinkers or recall bias and should be interpreted cautiously.

## Autoimmune Liver Diseases

This group of liver diseases includes autoimmune hepatitis (AIH), primary biliary cholangitis (PBC), and primary sclerosing cholangitis (PSC). As these are rare conditions, not much evidence regarding alcohol consumption is available, and results from studies in this area should be interpreted cautiously. In AIH, an inverse association between alcohol consumption and the diagnosis of AIH was seen in a dose-response pattern in a smaller study of 72 AIH patients [54]. For PBC, a case-control study of more than 2,500 PBC patients in the UK found that alcohol use, defined as if a person had ever consumed alcohol regularly, was negatively associated with the presence of PBC [55]. An association between any alcohol consumption and HCC was found in a smaller case-control study of 52 Chinese PBC patients with HCC, where alcohol consumption was more common in HCC cases (31%) than in controls (8%, *p* = .01) [56]. In a larger European study, however, no association was found between alcohol consumption over 40 g per day and HCC in 716 PBC patients [57].

In PSC, alcohol consumption has been suggested to increase the risk of development of cholangiocarcinoma (CCA) [58]. However, this study included only four PSC patients with CCA and current alcohol consumption, why the dose-response effect is unclear. In a study of 96 Swedish PSC patients, lifetime alcohol consumption was low at 2.6 units of alcohol per week in mean. No association between alcohol consumption and higher values on transient elastography was found [59]. No cases of CCA were included in this cross-sectional study.

## Hemochromatosis

Hereditary hemochromatosis (HH) is a genetic disorder of iron metabolism leading to inappropriate iron absorption and iron loading in various organs, especially the liver, and can lead to fibrosis progression and cirrhosis. In a study of 224 HH patients, 61% of subjects who consumed more than 60 g of alcohol per day had severe fibrosis or cirrhosis, compared to 7% of subjects who consumed less than 60 g per day [60]. Similar findings have been found in other studies [61–63]. The role of low to moderate alcohol consumption as a risk factor for fibrosis progression in HH remains to be studied.

## Liver Transplantation

ALD is one of the leading indications for liver transplantation (LTX), and cases listed for LTX due to ALD have increased by 45% between 2004 and 2013 in the USA [17]. Although no formal recommendation on alcohol consumption exist for non-ALD cases either from AASLD or EASL after LTX, [64, 65] a careful approach is often advocated. Indeed, around 60% of non-ALD cases continue to consume alcohol after LTX, although often in small amounts [66]. Excessive drinking after LTX, independent of the primary indication, is associated with increased mortality [67] and graft loss [68].

## Methodological Considerations

Several difficulties exist when trying to investigate the risk of alcohol consumption on disease progression or severity in concomitant liver disease. First, most studies investigating alcohol consumption have done so by using methodological designs including patients with manifest liver disease, including cirrhosis or HCC. This could induce misclassification bias of persons who has had a high lifetime consumption of alcohol but are currently abstaining due to symptoms, so-called “sick quitters” [69].

Also, patients under evaluation for any liver disease might be reluctant to disclose their true alcohol habits, or might unknowingly under-report these, leading to misclassification or recall bias. Indeed, in a recent study of 120 NAFLD patients who all reported drinking less than 14 units of alcohol per week, 11% of the sample had high levels of phosphatidyl ethanol (PEth) [28•], a validated marker for recent alcohol consumption [70–72], suggesting a higher than reported consumption of alcohol. This indicates that some patients with NAFLD could actually be classified as ALD patients. The use of validated biomarkers such as PEth could be one way to differentiate between NAFLD and ALD.

Second, different confounding factors could possibly explain the association between both detrimental and beneficial effects of alcohol and severity of liver disease. These include among others physical activity, smoking, dietary factors such as coffee and antioxidants, drinking patterns, and type of alcohol consumed, why such factors should ideally be accounted for in future studies.

Finally, genetic differences including mutations in the PNPLA3 [73, 74] and TM6SF2 genes [75, 76] are associated with increased risk of adverse outcomes in ALD and could influence the risk of disease progression that alcohol consumption might assert on any concurrent liver disease.

## Conclusions

In summary, alcohol consumption of more than 30 g per day in men and 20 g per day in women is associated with fibrosis progression, development of cirrhosis and hepatocellular carcinoma, and mortality in most liver diseases. Large, well-designed studies on the risk, or benefit, of low to moderate alcohol consumption in the setting of concurrent liver disease are missing but are eagerly awaited. An individual approach should be made by clinicians advising patients with known or suspected liver diseases.
